# Oral Propranolol Before 45 Weeks’ Corrected Gestational Age for Complicated Infantile Hemangiomas: A Retrospective Single-Center Study

**DOI:** 10.3390/children13101435

**Published:** 2026-10-19

**Authors:** Marco Pensabene, Maria Patti, Gregorio Serra, Fabio Baldanza, Francesco Grasso, Mario Giuffrè, Giovanni Licata Ricottone, Maria Sergio, Giovanni Corsello, Maria Rita Di Pace

**Affiliations:** 1 Pediatric Surgery Unit, Department of Health Promotion, Mother and Child Care, Internal Medicine and Medical Specialties “G. D’Alessandro”, University of Palermo, 90133 Palermo, Italy; 2 Neonatal Intensive Care Unit, Department of Health Promotion, Mother and Child Care, Internal Medicine and Medical Specialties “G. D’Alessandro”, University of Palermo, 90133 Palermo, Italy; 3 Pediatric and New-born Unit, Bressanone Hospital, Azienda Sanitaria dell’Alto Adige (SABES), Via Dante 51, 39042 Bressanone, Italy; giovanni.licataricottone@sabes.it

**Keywords:** infantile hemangioma, propranolol, premature infants, vascular tumors, beta-adrenergic blockers

## Abstract

**Highlights:**

**What are the main findings?**

- Oral propranolol offered promising results in terms of safety and effectiveness in our selected patients younger than 45 weeks of corrected gestational age.
- To increase tolerance to the drug and prevent adverse effects of propranolol, its administration in a controlled environment, such as neonatal intensive/semintensive care units, can be useful.

**What are the implications of the main findings?**

- Multicentric and prospective trials should investigate this topic to obtain robust evidence and consider extending recommendations for oral propranolol administration to this specific population.
- Neonatal intensive/semintensive care unit physicians and nurses should be familiar with this specific topic, since greater involvement of these units can be expected.

**Abstract:**

**Background**: Oral propranolol (OP) is the first-line treatment for complicated and high-risk infantile hemangiomas (IH). However, the optimal timing of treatment initiation in preterm infants remains uncertain, as current recommendations generally discourage administration before 45 weeks of corrected gestational age (CGA). We aimed to evaluate the safety and feasibility of early OP administration in selected preterm infants or term newborn with complicated high-risk IH. **Methods**: We retrospectively reviewed patients treated with OP for IH at a tertiary referral center between January 2023 and April 2026. Patients were divided according to CGA at treatment initiation into an early-treatment group (<45 weeks CGA) and a standard-timing group (≥45 weeks CGA). Clinical characteristics, treatment modalities, and adverse events were collected and compared between groups. **Results**: Seventy-five patients were included: 9 received early OP (mean CGA at treatment initiation 38 weeks + 3 days) and 66 that started treatment at or after 45 weeks CGA. Early-treated infants showed a higher prevalence of complicated IH at diagnosis (88.9% vs. 4.5%). No major adverse events, including clinically significant bradycardia, hypotension, arrhythmias, respiratory complications, or treatment discontinuation, occurred in either group. Transient asymptomatic hypoglycemia occurred in five patients, all belonging to the standard-timing group, and resolved with feeding. Treatment duration was comparable between groups. **Conclusions**: Early OP administration before 45 weeks CGA may represent a feasible and well-tolerated option in selected preterm infants and term newborn with complicated high-risk IH when initiated in a protected neonatal setting with careful monitoring. These findings may suggest individualized risk-based decision-making in selected cases, rather than routine treatment before current age thresholds.

## 1. Introduction

Infantile hemangiomas (IHs) are the most common benign vascular tumors of infancy, affecting approximately 4–5% of term infants and up to 23–30% of preterm newborns. Although most IHs are not fully evident at birth, they typically undergo a rapid proliferative phase during the first weeks of life, with nearly 80% of their final size reached by 5 months of age. In a minority of cases, particularly those involving deeper soft tissues, proliferation may continue until 9–12 months or even up to 24 months of age. While the majority of IHs regress spontaneously without requiring treatment, approximately 10–15% are considered high-risk because of their size, location, growth pattern, or associated complications, including ulceration, bleeding, functional impairment, or permanent disfigurement. Early recognition of these lesions is therefore essential to prevent irreversible functional and cosmetic sequelae [1,2,3,4,5].

The introduction of oral propranolol (OP) has revolutionized the management of IHs. Since its serendipitous discovery in 2008, propranolol has consistently demonstrated superior efficacy and a more favorable safety profile than previously available therapies, including systemic corticosteroids, interferon, vincristine, laser treatment, and surgery. By promoting early involution and arresting the proliferative phase, OP significantly reduces the risk of ulceration, functional compromise, and permanent esthetic sequelae. Consequently, current international guidelines recommend oral propranolol as the first-line treatment for complicated or high-risk IHs, and emphasize that therapy should be initiated as early as clinically indicated to maximize therapeutic benefit [1,6,7,8]. Despite these recommendations, uncertainty remains regarding the earliest safe timing for propranolol initiation. Current regulatory indications and clinical guidelines generally recommend starting treatment after 5 weeks of chronological age, or after 45 weeks of corrected gestational age (CGA) in preterm infants. These recommendations are largely based on the eligibility criteria adopted in pivotal clinical trials rather than on evidence demonstrating increased toxicity before this threshold. As a result, preterm infants with rapidly proliferating or complicated IHs may face a therapeutic dilemma: delaying treatment to comply with age recommendations may allow lesion progression and increase the risk of functional impairment or permanent tissue damage, whereas early treatment remains an off-label approach supported by limited evidence. Over the last few years, a small number of observational studies have reported encouraging safety and effectiveness data for propranolol initiated before the currently recommended age threshold in carefully selected infants. In a report by Gatts et al. in 2022 [9], the Authors report a retrospective analysis of adverse events and results of OP administered in a cohort of 24 patients with a mean age of 42 weeks of CGA. The Author found an acceptable profile of tolerability, with only minor events reported, finally advocating larger and prospective studies [8]. However, evidence remains scarce, particularly in premature newborns requiring treatment during neonatal hospitalization, and no clear criteria have been established to identify patients who may benefit from early therapy under close clinical monitoring [9,10].

The present study aimed to describe our single-center experience with early oral propranolol administration before 45 weeks corrected gestational age in preterm infants with complicated high-risk IHs. In addition, we compared their clinical characteristics and safety outcomes with those of infants who started treatment according to current recommendations, with the objective of further contributing to the limited evidence supporting individualized early treatment in selected neonatal patients.

## 2. Materials and Methods

### 2.1. Study Design and Population

We retrospectively reviewed all patients treated with oral propranolol (OP) for infantile hemangioma (IH) at our tertiary referral center between January 2023 and April 2026. Actual gestational age (GA) at birth was determined by the best obstetric estimate combining the last menstrual period and early booking ultrasound, and prematurity status was defined for infants born at <37 completed weeks of gestation. Chronological age (CA) at treatment initiation was defined as the time elapsed from birth to the first dose of OP (expressed in days). During the neonatal and early infancy period, postmenstrual age (PMA)—equivalently referred to in our methodology as corrected gestational age (CGA)—was calculated as the sum of gestational age at birth and chronological age (PMA = gestational age at birth + chronological age). For broader developmental or growth context, corrected age (CA) was calculated by subtracting the weeks of prematurity from chronological age, using the formula: CA = chronological age—(40—gestational age at birth). Patients were divided into two groups according to the corrected gestational age (CGA) at treatment initiation. Group A included patients who started OP before 45 weeks of CGA (early-treatment group), whereas Group B included patients who started OP at or after 45 weeks of CGA (standard-timing group).

Patients were considered eligible for OP treatment according to current international recommendations, including high-risk IHs and complicated lesions associated with ulceration, bleeding, functional impairment, rapid progression, or potential risk of permanent disfigurement.

Patients with clinical features suggestive of PHACE or LUMBAR syndrome were excluded from the study cohort in order to obtain a more homogeneous study population. Confirmation or exclusion of these syndromes may require MRI under sedation, together with specific cardiological assessment, including echocardiography, and additional imaging according to the clinical presentation. In our clinical setting, patients with suspected PHACE or LUMBAR syndrome were therefore scheduled for MRI under sedation of the relevant anatomical regions, cardiological assessment with echocardiography, and abdominal ultrasound. This diagnostic work-up could prolong the time required to complete the evaluation and consequently delay the initiation of propranolol. As a result, these patients were therefore excluded from the present analysis, although they continued to be managed according to standard clinical practice.

Patients with hypothyroidism were also excluded. In particular, thyroid dysfunction may occur in association with large or diffuse hepatic IH as part of consumptive hypothyroidism and may require specific diagnostic evaluation and, when indicated, thyroid hormone replacement therapy. This additional diagnostic and therapeutic pathway could similarly delay propranolol initiation. These patients were consequently not included in the present analysis, despite they were managed according to standard clinical practice.

### 2.2. Clinical and Treatment Assessment

For all included patients, demographic and clinical characteristics were collected, including gestational age at birth, birth weight, weight at OP initiation and completion, CGA at treatment onset, anatomical location of IH (head/neck, trunk, acral regions/extremities), lesion characteristics (superficial, deep, or mixed involvement), and the presence of complications. Among complications, functional impairment was defined as any clinically relevant impairment of the function of the involved anatomical site; suction impairment was considered a specific manifestation of functional impairment, referring to clinically evident impairment of sucking or feeding. Complication categories were not mutually exclusive, as more than one complication could occur in the same patient.

Treatment-related variables included age and setting of OP initiation (NICU/neonatal unit hospitalization or outpatient setting), starting dose, time required to reach the target dose, duration of therapy, and final therapeutic dosage.

In all patients, OP was administered at a target dose of 3 mg/kg/day divided into two daily administrations. Dose escalation was performed gradually, starting from 0.5 to 1 mg/kg/day with subsequent weekly increases according to clinical tolerance, so that the therapeutic dosage was reached within the first month of OP administration. In infants receiving early treatment before 45 weeks of CGA, the initial dose was generally reduced to 0.5 mg/kg/day to allow a more gradual adaptation to beta-blockade.

During treatment initiation and dose escalation, patients underwent clinical monitoring including heart rate, blood pressure, oxygen saturation, and blood glucose assessment. After reaching the target dose, patients were evaluated monthly during follow-up. The monitoring protocol was designed as follows. Vital parameters were assessed at predefined time points. Before treatment initiation, heart rate, blood pressure, oxygen saturation, and blood glucose were assessed during the first clinical evaluation and again during the electrocardiographic/cardiological assessment preceding the initiation of therapy.

All parameters were assessed immediately before the first OP dose, usually at 8.00–8.30 AM, after at least 3 h from the last meal. Following the first administration and each subsequent dose escalation, patients underwent structured clinical observation for a duration of 4 to 6 h. Vital parameters (heart rate, blood pressure, oxygen saturation) and blood glucose were systematically assessed pre-dose and at strict intervals (1, 2, 3, and 4 h post-dose); during this period all patients were clinically monitored.

This monitoring scheme was repeated weekly during the first month and during the subsequent dose increases, and then monthly once the dose had been adjusted according to body weight.

Bradycardia, hypotension, and hypoglycemia were defined according to international consensus guidelines for propranolol use in infancy: specifically, bradycardia was defined as a persistent heart rate <100 bpm for infants aged <3 months (or a clinically significant reduction >20% from baseline), hypotension as a mean arterial pressure below the age-adjusted 10th percentile, and hypoglycemia as a blood glucose level <45 mg/dL (2.5 mmol/L) [11,12].

Treatment tolerability was assessed based on the occurrence of adverse events according to predefined criteria. Adverse events were evaluated collegially by the physicians involved in patient care at the time of occurrence and subsequently documented in clinical records. Events were classified as major adverse events, defined as unexpected clinical conditions requiring hospital admission or discontinuation of propranolol (e.g., severe hypoglycemia, clinically significant bradycardia, hypotension, arrhythmias, or respiratory compromise), and minor adverse events, defined as conditions that could be successfully managed without hospitalization and either resolved spontaneously with adaptation to treatment or were managed by adjusting propranolol administration (e.g., restlessness, sleep-related disturbances, and cold extremities). Cold extremities were considered a possible manifestation of peripheral vasoconstriction associated with beta-adrenergic blockade.

### 2.3. Outcomes and Statistical Analysis

The primary objective was to evaluate the safety profile of early OP administration, focusing on the occurrence of major adverse events. Secondary outcomes included treatment tolerance, duration of therapy, and clinical evolution of IH. Clinical characteristics and outcomes were compared between groups and with previously published data from the literature.

Continuous variables were reported as mean values and ranges due to the limited sample size and non-normal distribution of data. Comparisons between groups were performed using the Mann–Whitney U test. Categorical variables, including adverse events and anatomical distribution of lesions, were compared using Fisher’s exact test. Statistical significance was defined as a *p* value < 0.05.

## 3. Results

### 3.1. Study Population and Treatment Characteristics

During the study period, 184 infants were referred to our institution for specialized evaluation of infantile hemangioma (IH). Among them, 109 patients were excluded because oral propranolol (OP) treatment was not indicated (91 patients with superficial/low-risk IH) or because they were older than 9 months, when the lesion growth has reached a plateau, and thus the patient is within a suboptimal window for treatment initiation, according to guidelines (17 patients) [1]. One additional patient has been excluded because of congenital hypothyroidism diagnosis delayed OP initiation.

Overall, 75 patients were included in the study. Nine infants started OP before 45 weeks of corrected gestational age (CGA) and were assigned to Group A (early-treatment group), whereas 66 infants started treatment at or after 45 weeks of CGA and were assigned to Group B (standard-timing group).

Baseline characteristics of the study population, treatment-related variables, and clinical features of IH are summarized in Table 1. The mean CGA at OP initiation was 38 weeks + 3 days (range 35 weeks + 3 days–40 weeks + 6 days) in Group A and 49 weeks + 4 days (range 45–68 weeks) in Group B. The mean duration of OP therapy was comparable between groups, with 10 months (range 8–12 months) in Group A and 10.5 months (range 7–13 months) in Group B (*p* = 0.62, Mann–Whitney U test). Weight at treatment initiation was significantly lower in Group A compared with Group B (mean 2380 g, range 1800–3500 g vs. 5010 g, range 4700–5200 g; *p* < 0.001). Similarly, weight at treatment completion was lower in Group A compared with Group B (mean 8.8 kg, range 7–10 kg vs. 11.25 kg, range 9.6–12.5 kg; *p* < 0.001).

### 3.2. Clinical Characteristics of Infantile Hemangiomas

The anatomical distribution and clinical characteristics of IH are summarized in Table 1. In Group A, IH involved the head and neck region in 3 patients (33.3%), the trunk in 2 patients (22.2%), and acral regions/extremities in 3 patients (33.3%). In all these cases, a mixed involvement of superficial and deep tissues was observed. One infant presented with multifocal IH involving 13 lesions (Table 2).

In Group B, IH involved the head and neck region in 45 patients (68.2%), the trunk in 13 patients (19.7%), and acral regions/extremities in 8 patients (12.1%). A mixed superficial/deep involvement was observed in 51 patients (77.3%).

**Figure 1:**
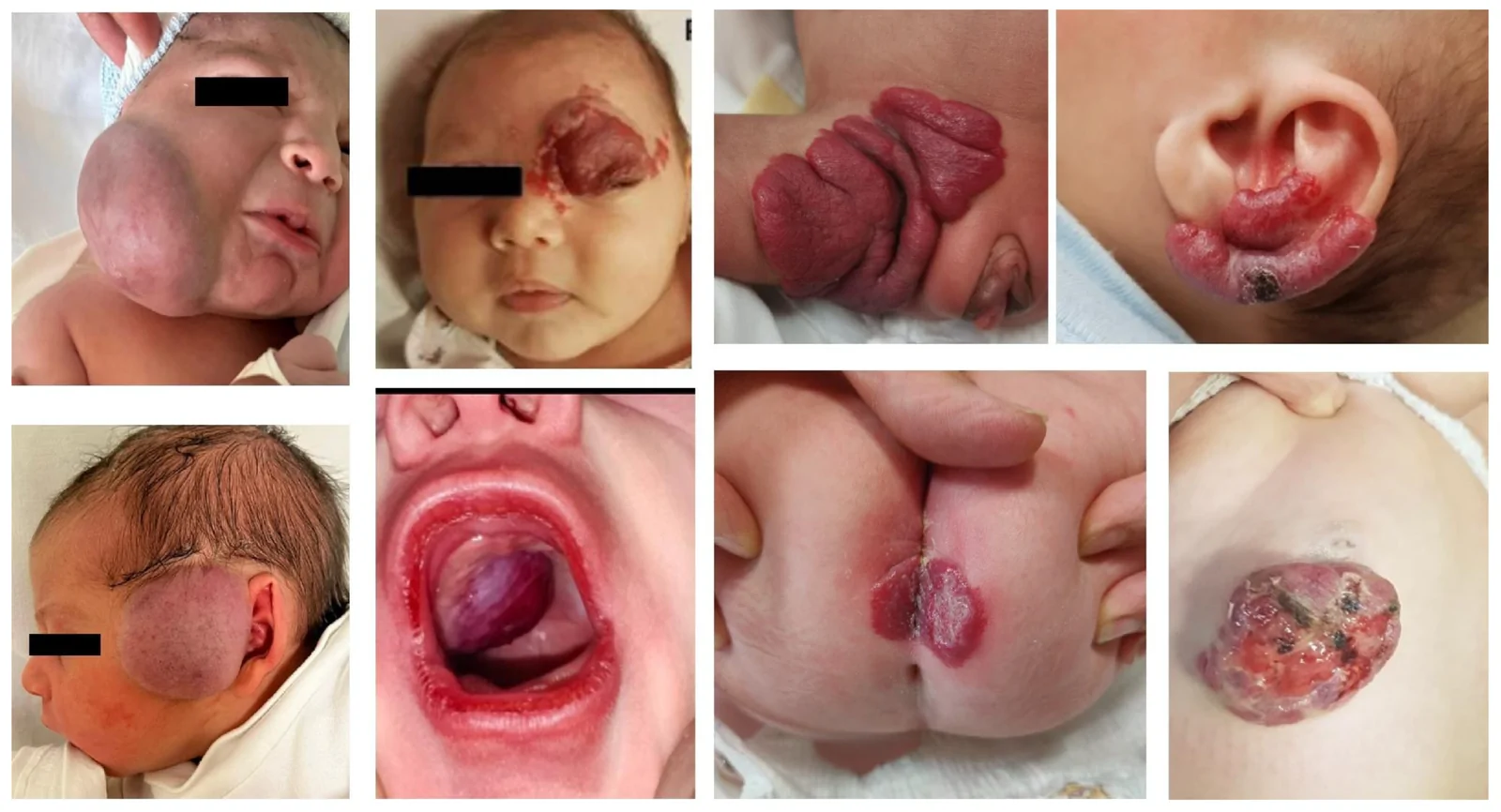
The picture illustrates complicated lesions occurring in the described patients: ulceration and functional impairment can be observed.

Overall, 11 patients (14.7%) presented with complicated IH at diagnosis (Figure 1). Complicated lesions were observed in 8/9 patients (88.9%) in Group A and in 3/66 patients (4.5%) in Group B.

Ulceration and/or bleeding represented the most frequent complication, occurring in 6 patients in Group A and 3 patients in Group B (Figure 1). Functional impairment was observed in 5 patients in Group A and 2 patients in Group B. One infant in Group A presented with an ulcerated and bleeding palatal IH associated with severe anemia requiring blood transfusion.

### 3.3. Treatment Setting and Safety Outcomes

The setting of OP initiation differed between groups (Table 1). All infants in Group A received the first OP administration during hospitalization in the NICU or Neonatal Unit, whereas all patients in Group B started treatment in an outpatient setting.

Safety outcomes during OP treatment are summarized in Table 3. No major adverse events related to OP administration were observed in either group (Group A: 0/9, 0%, 95% CI 0–33.6%; Group B: 0/66, 0%, 95% CI 0–5.4%). In particular, no clinically significant bradycardia, hypotension, arrhythmias, respiratory complications, or treatment discontinuation due to intolerance occurred. Transient asymptomatic hypoglycemia (blood glucose <45 mg/dL) occurred in five patients, all belonging to Group B. All episodes occurred before 4 months of age and resolved after feeding without pharmacological intervention. Seven patients in Group B (10.6%) experienced restlessness or sleep-related disturbances (insomnia) during treatment; symptoms resolved after dividing the daily dose into three administrations. No cases of cold extremities were reported.

**Figure 2:**
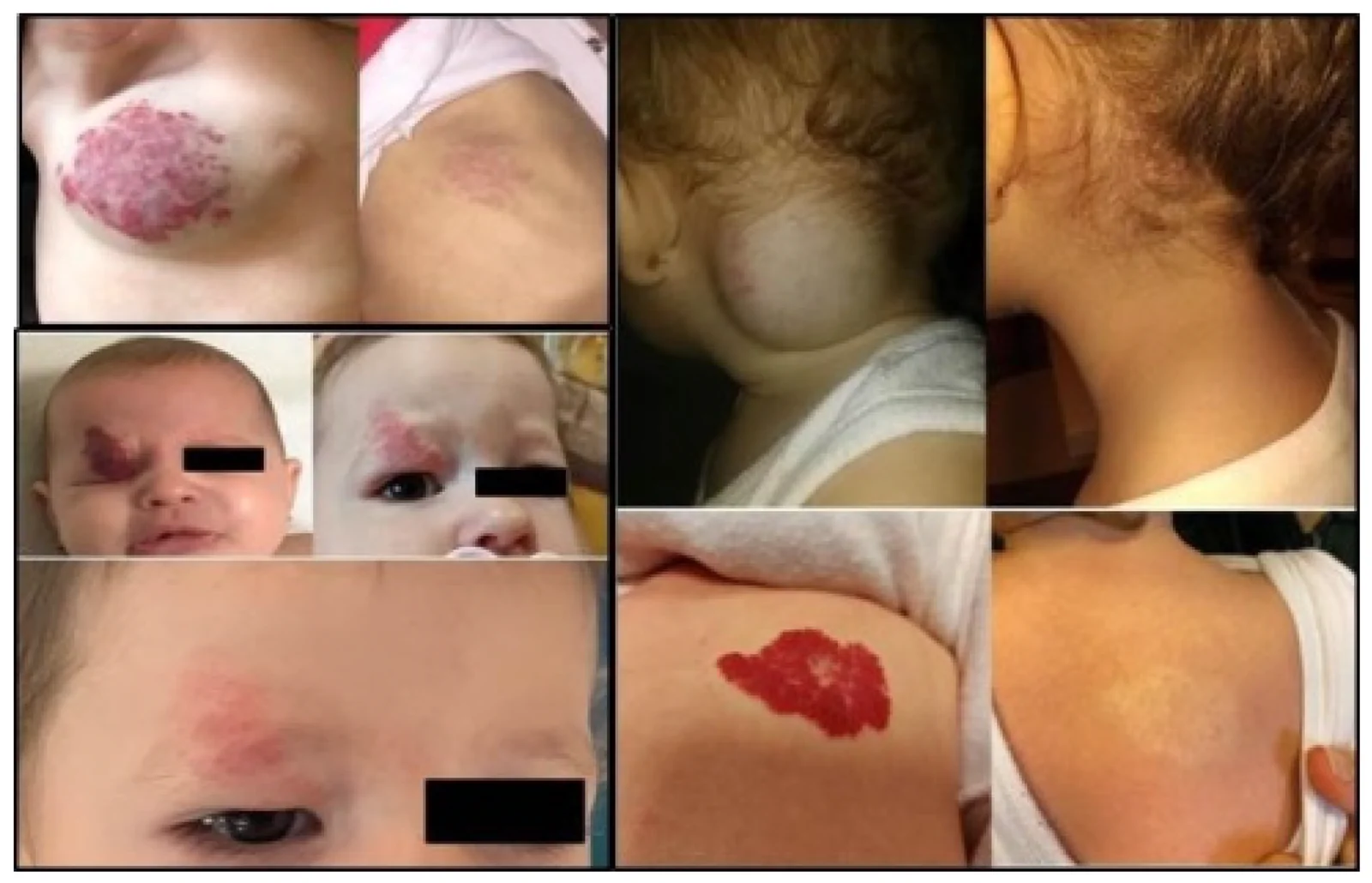
The figure shows the lesions before OP administration and at the end valuable results of the therapy in the same patients.

**Figure 3:**
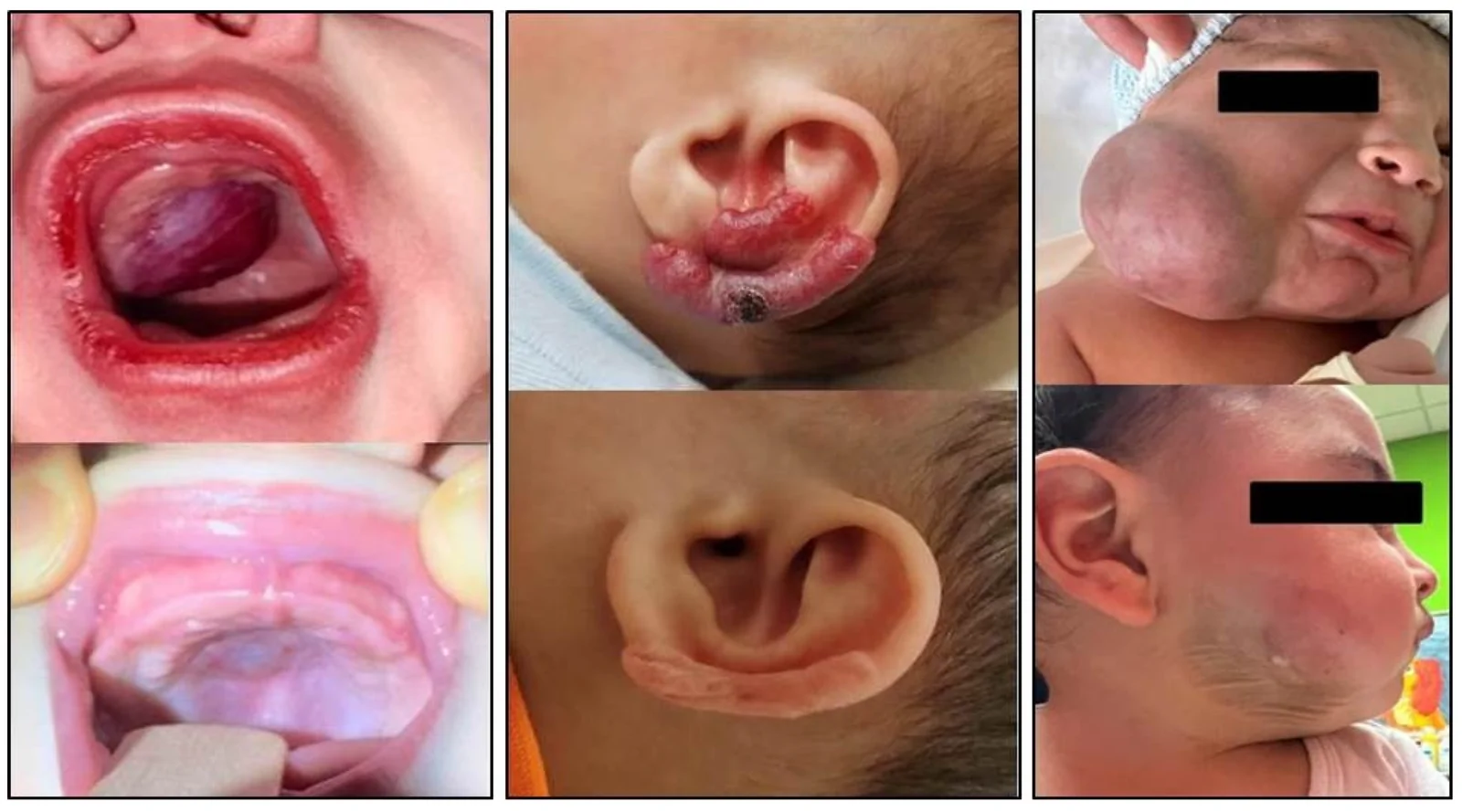
The figure shows the evolution of the lesions occurring in 3 patients in A group (OP before 45 W of CGA) after OP administration. Despite the important result and the involution of the lesions, little fibro–fatty remnants can be observed in two patients.

All patients completed the planned treatment course, and no patient required permanent discontinuation of OP because of adverse effects. In all patients, a clinical improvement/involution was observed during follow-up (Figure 2 and Figure 3).

## 4. Discussion

The present study describes our experience with early oral propranolol (OP) administration before 45 weeks of corrected gestational age (CGA) in a selected cohort of preterm infants with complicated high-risk infantile hemangiomas (IH). In our series, early treatment was associated with a favorable safety profile, with no major cardiovascular, respiratory, or metabolic adverse events and no treatment discontinuations due to intolerance. These findings suggest that, in carefully selected infants and within an appropriate monitoring setting, OP administration before the currently recommended age threshold may represent a feasible therapeutic option when the potential risks of delaying treatment exceed the theoretical risks of early beta-blockade.

Since the first report of propranolol efficacy in IH, the management of these vascular tumors has dramatically changed. Compared with previous therapeutic strategies, including systemic corticosteroids, interferon, vincristine, laser therapy, and surgery, OP provides rapid clinical response, ease of administration, and a favorable safety profile, becoming the first-line treatment for complicated and high-risk IH [6,7,8,13,14,15,16,17,18,19,20,21,22]. However, uncertainty remains regarding the earliest safe timing for treatment initiation, particularly in preterm and newborn infants. Current recommendations generally discourage OP administration before 5 weeks of chronological age or before 45 weeks of CGA, mainly because infants below these thresholds were excluded from pivotal clinical trials and because of concerns regarding hypoglycemia, cardiovascular instability, and respiratory adverse events in this vulnerable population [1].

In our cohort, nine infants received OP before 45 weeks of CGA, with treatment initiated at a mean CGA of 38 weeks + 3 days. This represents one of the earliest reported treatment timings among available studies [9,10,11]. Previous reports have suggested that early propranolol administration in selected newborns may be safe and effective, including infants treated before five weeks of corrected age [9,10,23,24,25,26]. However, available evidence remains limited, consisting mainly of retrospective studies and small cohorts. Our findings add further real-world data supporting the feasibility of early OP treatment in a highly selected neonatal population. Importantly, infants receiving early treatment in our study represented a subgroup with a greater clinical burden. Eight of nine patients in Group A presented with complicated IH at diagnosis, compared with three of sixty-six patients in Group B. This difference reflects the clinical rationale underlying early treatment decisions: infants requiring OP before 45 weeks of CGA are not simply younger patients with uncomplicated lesions, but rather patients in whom rapid progression, ulceration, bleeding, functional impairment, or risk of permanent damage may justify an individualized therapeutic approach. In these circumstances, postponing treatment until the conventional age threshold may expose infants to preventable complications, whereas the potential risks associated with propranolol can be minimized through careful monitoring.

The main concern regarding early OP administration is cardiovascular tolerance. Beta-adrenergic blockade may theoretically lead to bradycardia, hypotension, or impaired cardiovascular adaptation, particularly in preterm infants with immature autonomic regulation. In our cohort, no clinically significant bradycardia, hypotension, arrhythmias, or respiratory complications were observed. These findings are consistent with previous reports suggesting that propranolol can be safely administered in young infants without pre-existing cardiovascular contraindications when appropriate monitoring is ensured [1,9,10]. Hypoglycemia represents another major concern, especially in premature infants because of limited glycogen stores, immature counter-regulatory mechanisms, and potential feeding difficulties. Interestingly, all five episodes of transient asymptomatic hypoglycemia observed in our cohort occurred in Group B, and none occurred among infants treated before 45 weeks of CGA. These events resolved after feeding and did not require pharmacological intervention. Although the small sample size prevents definitive conclusions, this observation suggests that the risk of hypoglycemia is influenced not only by biological maturity but also by treatment management, feeding schedules, and caregiver education.

In our setting, early-treated infants received inpatient monitoring with scheduled feeding and close clinical surveillance, which may have contributed to early recognition and prevention of metabolic complications [27,28]. Parental education and advice could play an important role in the safety profile of propranolol, and thus, risks of hypoglycemia, bradycardia, and hypotension should be familiar to parents and caregivers. Other minor adverse events were uncommon and manageable. Restlessness or sleep-related disturbances occurred only among infants in Group B and resolved after dividing the daily dose into three administrations. No cases of cold extremities or treatment discontinuation were recorded. Overall, our findings support previous evidence indicating that most OP-related adverse effects are transient, reversible, and rarely treatment-limiting when patients are appropriately selected and families are adequately educated, as it has been shown in other reports and in other fields of applications [23,24,25,26,29,30]. To this point, the experience of Ji et al. added a promising perspective. In a cohort of newborns prospectively evaluated and treated with OP, a significant reduction in heart rate was observed, but this condition was self-limiting and not associated with reduced diastolic blood pressure or blood glucose, suggesting a good tolerability profile of OP, in selected newborn patients. In another study, the Author reported a low prevalence of intolerable side effects of OP, suggesting that low birth weight and age should be considered predictive of an increased risk for intolerable side effects [23,24,25,30].

A relevant aspect of our experience is the setting in which early OP was initiated. All infants in Group A received their first doses during NICU or Neonatal Unit hospitalization, allowing repeated assessment of heart rate, blood pressure, oxygen saturation, and glucose levels. Furthermore, neonatal staff are experienced in identifying subtle signs of clinical deterioration and promptly addressing feeding intolerance, hypoglycemia, or cardiorespiratory instability. This controlled environment likely represents an essential component of early treatment safety and should be considered whenever OP is initiated outside current age recommendations. Another possible contributor to treatment tolerability was the cautious dose escalation adopted in our cohort. Rather than immediately starting with 1 mg/kg/day, therapy in early-treated infants was initiated at 0.5 mg/kg/day and progressively increased to the target dose of 3 mg/kg/day. This gradual approach may allow better adaptation to beta-blockade and facilitate early identification of intolerance, particularly in low-weight infants or those with uncertain nutritional intake.

Early OP administration should not be interpreted as a routine approach for all high-risk IH. Current recommendations remain appropriate for the majority of patients, and many lesions can be safely monitored before treatment initiation, while others should be carefully managed, given the increased risk of severe complications [31,32]. Our findings instead emphasize the importance of individualized decision-making based on lesion characteristics, clinical evolution, and the balance between the potential harm of delayed treatment and the manageable risks of early therapy. Early OP may therefore be considered in selected infants with rapidly progressive, ulcerated, bleeding, function-threatening, or potentially disfiguring IH, following multidisciplinary evaluation and initiation in a setting capable of adequate monitoring and long follow-up, as required in newborn and pediatric diseases [33,34,35,36,37].

In all patients, OP treatment resulted in clinical improvement, although formal volumetric assessment or standardized scoring systems were not applied; in all patients, the lesion involution was documented through serial clinical photographs obtained throughout the treatment course and acquired according to standardized conditions, including lighting, patient positioning, and camera-to-patient distance, with the aim of ensuring the most consistent and reproducible visual assessment. Nevertheless, objective comparisons of treatment response between groups were not possible, representing an additional limitation of the study. Nevertheless, all infants completed therapy, and treatment duration was comparable between groups, suggesting that earlier initiation did not require prolonged administration to control the proliferative phase of IH. This study has several limitations. First, its retrospective design exposes the results to potential selection bias and incomplete standardization. In addition, the two groups include patients with different clinical characteristics, which limits the reproducibility of the comparison. Second, the early-treatment cohort was small, reflecting the rarity of infants requiring intervention before 45 weeks of CGA, and limiting the statistical power of comparisons between groups. Third, this was a single-center study performed in a tertiary neonatal setting with specific expertise in monitoring fragile infants; therefore, results may not be directly applicable to centers with fewer neonatal resources. Despite these limitations, our study provides additional real-world evidence regarding an understudied clinical scenario. The inclusion of a comparison group treated according to current recommendations, together with the detailed safety assessment, could represent a strength of the study, despite the difference in group population. Importantly, the favorable outcomes observed in a cohort composed mainly of infants with severe presentations support the concept that treatment decisions should be individualized according to clinical risk rather than based exclusively on chronological thresholds.

## 5. Conclusions

Our experience suggests that early OP administration may be a feasible and well-tolerated option in selected preterm infants with complicated, high-risk IH when treatment is initiated in a protected neonatal setting, with gradual dose escalation and careful monitoring of cardiovascular and metabolic parameters. These findings do not support routine treatment before the currently recommended age thresholds but rather emphasize the importance of individualized decision-making based on lesion severity, clinical evolution, and multidisciplinary risk assessment. Further prospective multicenter studies are needed to better define selection criteria and confirm the safety profile of early OP administration in this vulnerable population.

## Figures and Tables

**Table 1:** Baseline characteristics and clinical features of patients treated with oral propranolol according to timing of treatment initiation.

Characteristics	Group A: Early Treatment (<45 Weeks CGA) (*n* = 9)	Group B: Standard Timing (≥45 Weeks CGA) (*n* = 66)
Gestational age at birth, weeks, mean (range)	32 weeks + 3 days (29 + 0–39 + 2)	39 weeks + 2 days (38 + 1–41 + 3)
Birth weight, g, mean (range)	1719 g (770–3120)	3090 g (2640 –3550)
CGA at OP initiation, mean (range)	38 weeks + 3 days (35 + 3–40 + 6)	49 weeks + 4 days (45–68)
Weight at OP initiation, g, mean (range)	2380 (1800–3500)	5010 (4700–5200)
Weight at treatment completion, kg, mean (range)	8.8 (7–10)	11.25 (9.6–12.5)
Treatment duration, months, mean (range)	10 (8–12)	10.5 (7–13)
**IH location, *n* (%)**	/	/
Head and neck	3 (33.3%)	45 (68.2%)
Trunk	2 (22.2%)	13 (19.7%)
Acral regions/extremities	3 (33.3%)	8 (12.1%)
Multifocal IH	1 (11.1%)	0
**Complicated IH at diagnosis, *n* (%)**	8 (88.9%)	3 (4.5%)
Ulceration and/or bleeding	6 (66.7%)	3 (4.5%)
Functional impairment	5 (55.%)	2 (3.0%)
Severe anemia requiring transfusion	1 (11.1%)	0
**First OP administration setting, *n* (%)**	/	/
NICU/Neonatal Unit hospitalization	9 (100%)	0
Outpatient setting	0	66 (100%)

**Table 2:** Characteristics of Group A patients with complicated lesions.

Patients	Sex	District Involved	Site of the Lesion	Complications Occurred
1	M	Head and neck	Face (Mandibula)	Functional (suction) impairment/disfigurement
2	M	Head and neck	Face (Parothid)	Functional (suction) impairment/disfigurement
3	M	Head and neck	Hard Palate	Ulceration, bleeding-anemia/Functional (suction) impairment
4	F	Acral/Extremities	Hand	Functional impairment/ulceration
5	M	Acral/Extremities	Ear	Ulceration
6	F	Acral/Extremities	Finger	Functional impairment/Ulceration
7	F	Trunk	Abdomen	Ulceration
8	F	Trunk	Groin, female genitalia	Ulceration
9	M	Multiple	Multiple	Disfigurement (potential)

**Table 3:** Adverse effect reported during oral propranolol treatment.

Safety Outcomes	Group A: Early Treatment (<45 Weeks CGA) (*n* = 9)	Group B: Standard Timing (≥45 Weeks CGA) (*n* = 66)
Any adverse event, *n* (%)	0	12 (18.2%)
Severe adverse events, *n* (%)	0	0
Severe hypoglycemia requiring intervention	0	0
Transient asymptomatic hypoglycemia (<45 mg/dL)	0	5 (7.6%)
Bradycardia	0	0
Hypotension	0	0
Arrhythmias	0	0
Respiratory complications/desaturation	0	0
Restlessness/sleep-related disturbances	0	7 (10.6%)
Cold extremities	0	0
Treatment discontinuation due to adverse effects	0	0

## Data Availability

The data presented in this study are available on specific request from the corresponding authors due to privacy reasons.

## Abbreviations

The following abbreviations are used in this manuscript:

CGA	Corrected gestational age
IH	Infantile Hemangiomas
NICU	Neonatal Intensive Care Unit
OP	Oral Propranolol
